# Oxidative Stress in Aquatic Organisms

**DOI:** 10.3390/antiox12061223

**Published:** 2023-06-06

**Authors:** Changyou Song, Cunxin Sun, Bo Liu, Pao Xu

**Affiliations:** 1 Key Laboratory of Freshwater Fisheries and Germplasm Resources Utilization, Ministry of Agricultural and Rural Affairs, Freshwater Fisheries Research Center, Chinese Academy of Fishery Sciences, Wuxi 214081, China; songchangyou@ffrc.cn (C.S.); suncx@ffrc.cn (C.S.); 2 Wuxi Fisheries College, Nanjing Agricultural University, Wuxi 214081, China

Oxidative stress mainly refers to the imbalance between reactive oxygen species production and antioxidant defense systems in organisms [1]. Excessive oxidative stress can induce cell and tissue damage, mainly manifested as DNA hydroxylation, protein denaturation, lipid peroxidation and cell apoptosis [2]. In the past few decades, aquaculture has developed rapidly and has become the fastest growing food production sector for humans. Oxidative stress is ubiquitous in aquatic animals. In general, endogenous and exogenous factors are the main elements that induce oxidative stress, including temperature, oxygen, life history, nutrition, food deprivation, and industrial and agricultural pollutants [3]. In accordance with other animals, aquatic organisms have evolved complex mechanisms to resist oxidative stress. Therefore, uncovering the causes of oxidative stress, elucidating the underlying physiological mechanisms and developing antioxidant strategies are of great importance for the development of aquaculture.

In this Special Issue, 28 original scientific research papers are published, all of which highlight the most recent advances in all aspects of oxidative stress response in aquatic animals, including the generation process, the response mechanism and the resistant approaches. Papers in this Special Issue provide an updated overview of the advances in oxidative stress research, with advanced molecular approaches in both living organisms and habitats of aquatic animals.

Taxonomically, these papers cover freshwater fish (*Carassius auratus* [4], *Monopterus albus* [5], *Ictalurus punctatus* [6], *Megalobrama amblycephala* [7], *Micropterus salmoides* [8], *Lateolabrax maculatus* [9,10,11,12], *Carassius gibelio* [13,14], *Aplodinotus grunniens* [15], *Pangasianodon hypophthalmus* [16], *Danio rerio* [17], and hybrid grouper [18]), marine fish (*Scophthalus maximus* [19]), crustaceans (*Macrobrachium rosenbergii* [20,21,22], *Litopenaeus vannamei* [23,24,25], *Penaeus monodon* [26], *Scylla paramamosain* [27], and *Eriocheir sinensis* [28]), and molluscs (*Crassostrea hongkongensis* [29], *Trachinotus ovatus* [30], and *Pacific abalone* [31]). Meanwhile, these papers reveal several endogenous and exogenous factors that induce oxidative stress, such as environmental factors (water hardness [4], chronic hyperthermia [6], acute hypoxic stress [10], acute ammonia nitrogen [12], hypothermia [15], low salinity [23], and ammonia-N-stress [26]), nutritional factors (high carbohydrate levels [5], oxidized lipids [7], high-fat diet [12,20], and lipopolysaccharide [29,31]), essential or heavy metals (Zn [4], cadmium [13,27], Cu^2+^ [28], and polyinosinic–polycytidylic acid sodium salt [31]), pathogenic bacteria or virus (aflatoxin B1 and cyprinid herpesvirus 2 [14], water bubble disease (WBD) [21], *Vibrio harveyi* [29,31], and *Streptococcus agalactiae* [30]), and feeding practices (stocking density [8], transport stress [11]). Therapeutically, some papers have also explored some medicines or immunostimulants to resist oxidative stress, such as herbal medicine (mulberry leaf flavonoids [5], emodin [7], berberine [17], *Sophora flavescens* root extract [19], and tea tree oil [22]), nutritional stimulants (*Atractylodes macrocephala* polysaccharide [9], taurine alleviates [13], histamine [16,18], vitamin E [20], β-Glucan [23], krill oil [24], and zinc [25]), antibiotics (florfenicol and ofloxacin [21]), and feeding administration (feed restriction [6]). Additionally, the full length of antioxidant genes was also cloned to analyze their functional responses to oxidative stress, such as glutaredoxin [26] and catalase [31].

To uncover the relationship between oxidative stress inducers and response mechanisms, tissue or cell morphology, antioxidant and immunity enzymes and related gene expression, metabolic homeostasis, and cell fate related autophagy and apoptosis, as well as mortality, have been extensively studied in these papers. Mechanically, some key molecular signaling processes were studied, such as Nrf2 signaling [7,9,22,27], Notch signaling [7], PPAR signaling [8], MAPK signaling [10], NF-κB signaling [15,20,30], Relish-Imd signaling [22], TLR2-MyD88-NF-κB [28], and epigenetic regulator m6A methylation [17]. Additionally, the regulation of gut microbiota on oxidative stress resistance was also investigated in some papers [5,10,11,14,24,25]. A brief summary of each paper is shown below for reference.

Specifically, in freshwater fish, Choi et al. [4] investigated the toxicity stress of Zn and water hardness in *C. auratus*. They found that high water hardness can influence the absorption of Zn, and alleviating the hardness levels can reduce the toxicity stress caused by Zn. Shi et al. [5] found that 300 mg/kg mulberry leaf flavonoids (MLF) can alleviate the negative effects of high-carbohydrate-induced low growth performance, glucose metabolism disorder, liver oxidative damage and intestinal microbiota disturbance in *M. albu*, and that the relief of MLF is dose-related. Lu et al. [6] investigated the effects and mechanisms of feed restrictions on improving chronic, heat-induced (27 to 31 °C) liver peroxidation and damages in channel catfish (*I. punctatus*). They concluded that 2.5% body weight/day is recommended to improve antioxidant capacity and liver health during the summer season. Song et al. [7] studied the therapeutic mechanism of emodin on metabolic and oxidative disorders induced by dietary oxidized fish oil in *M. amblycephala* liver. Their results indicate that oxidative stress blocked the crosstalk between Notch and Nrf2 signaling, while emodin rescued Notch-Nrf2 interaction to ameliorate oxidative stress. The crosstalk between Notch and Nrf2 signaling might be the potential therapeutic target for emodin to ameliorate oxidative stress and metabolic disorder in the liver. Jia et al. [8] focused on the effects of stocking density on fish health in integrated rice–fish farming systems. They indicate that a high density (HD, 120 g/m^3^) inhibited growth and caused physiological responses, oxidative stress, and abnormal hepatic lipid metabolism in *M. salmoides*. Dong et al. [9] revealed that 400–4000 mg/kg *Atractylodes macrocephala* polysaccharide (AMP) could improve growth performance and antioxidant activity, as well as nutrient absorption in largemouth bass. Specifically, Nrf2 signaling was involved in the regulation. Song et al. [10] showed that hypoxia caused oxidative stress, exfoliation of the intestinal villus epithelium, and villus rupture, and increased cell apoptosis in largemouth bass (*M. salmoides*). Mechanically, MAPK signaling pathway and inflammatory related microbiota played an important role under hypoxic stress. Wang et al. [11] indicated that transport stress resulted in oxidative stress, and altered innate immune responses and affected the gut microbial compositions, mainly among proteobacteria, firmicutes, cyanobacteria and spirochaetes in juvenile largemouth bass. Dong et al. [12] revealed that the potent endoplasmic reticulum stress (ERs) inhibitor 4-PBA could decrease the peroxidation content and attenuated ERs induced by high-fat diet (HFD) and acute ammonia nitrogen. Xu et al. [13] revealed that taurine (1%) alleviates cadmium-induced endoplasmic reticulum stress via autophagy and apoptosis in gibel carp (*C. gibelio*), demonstrating the potential use of taurine in the mitigation of heavy metal toxicity in aquatic organisms. Xue et al. [14] investigated the defensive ability of gibel carp exposed to aflatoxin B1 (AFB1) by challenging it with cyprinid herpesvirus 2 (CyHV-2) infection. Their results indicate that AFB1 may increase the susceptibility of *C. gibelio* to CyHV-2 infection, and thus amplify the viral outbreak to endanger ecological safety in an aquatic environment. Chen et al. [15] revealed that acute hypothermia (10 °C for 8 d) induced oxidative stress, immunosuppression, mitochondrial enlargement, nucleoli aggregation, lipid droplet accumulation, metabolism, programmed cell death, and disease. Interactively, apoptosis and inflammation in immune organs were correlated with antioxidation and immunity suppression induced by hypothermia exposure. Liu et al. [16] indicated that high dietary histamine (480 mg/kg) decreases intestinal immunity and antioxidant capacity, inducing digestive tract oxidative damage and ultimately decreasing the growth of striped catfish (*P. hypophthalmus*). Zhang et al. [17] revealed that Berberine (BBR) ameliorates cellular oxidative stress, apoptosis and autophagy induced by lipid metabolism disorder by mediating Camk1db m6A methylation through the targeting of the Camk1db/ERK pathway in zebrafish-hepatocyte. Zhang et al. [18] revealed that high levels of histamine (≥404.12 mg/kg) were detrimental to the digestive physiology function and muscle quality of hybrid grouper (*Epinephelus fuscoguttatus* ♀ × *Epinephelus lanceolatus* ♂), although it did compromise its growth performance.

Accordingly, in marine fish, Hou et al. [19] indicated that 0.1–0.2% *Sophora flavescens* root extract (SFE) improved the growth performance, antioxidant activity and disease resistance against *Edwardsiella tarda* in *S. maximus*.

Moreover, in crustaceans, Sun et al. [20] found that 600 mg/kg dietary vitamin E alleviated hepatopancreas oxidative stress and apoptosis induced by high fat diet (13% dietary lipid in the diet) in *M. rosenbergii*, and that the NF-κB/NO signaling pathway was the antioxidant target for oxidative stress. Zhao et al. [21] demonstrated how antibiotic florfenicol and ofloxacin prevent *Citrobacter freundii*-induced water bubble disease (WBD) in giant freshwater prawns, *M. rosenbergii*, evidenced by improved antioxidant capacity, immune-related gene expression, and the anti-lipopolysaccharide factor in hepatopancreas. Liu et al. [22] reported that 100 mg/kg tea tree oil (TTO) could alter the hepatopancreatic lipid metabolism by affecting the antioxidant–autophagy axis in *M. rosenbergii*. The relish-Imd pathway functions significantly in the regulation. Qiao et al. [23] suggested that dietary β-glucan (0.1–0.2%) markedly increased growth performance and alleviated the negative effects of low-salinity stress by contributing to the activity of biochemical enzymes and enriching carbohydrate metabolism. Liang et al. [24] indicated that Krill oil is a suitable dietary phospholipid source to improve antioxidant capacity and innate immunity and establish the intestinal immune barrier by increasing the richness of Fusibacter, promoting the growth of Pacific white shrimp *L. vannamei*. Yang et al. [25] revealed that organic zinc had a higher bioavailability to improve zinc homeostasis, antioxidant capacity, immune response, glycolysis and intestinal microbiota, and was therefore a more beneficial zinc resource than inorganic zinc in white shrimp (*L. vannamei* Boone, 1931). Fan et al. [26] identified a novel glutaredoxin (PmGrx2) in *P. monodon*. They cloned the full length and indicated that PmGrx2 is involved in redox regulation and plays an important role in resistance to ammonia-N-stress. Cheng et al. [27] revealed that cadmium exposure increased H_2_O_2_ production, lipid peroxidation and tissue damage in mud crabs, but decreased the activity of SOD and catalase CAT, and caused lipid peroxidation and tissue damage. With Nrf2 knockdown, antioxidant capacity was decreased, leading to aggravated hepatotoxicity and cell injury. Feng et al. [28] revealed that Cu^2+^ exposure decreased antioxidative capacity and immunity, promoted lipid peroxidation and induced apoptosis, autophagy and ER stress in Chinese mitten crab (*E. sinensis*); the toxicity may be implicated following the activation of the ERK, AMPK, and TLR2-MyD88-NF-κB pathways.

Additionally, in molluscs, Ma et al. [29] suggested that post-spawning-phase male Hong Kong oysters *C. hongkongensis* have a more significant energy metabolic response and a greater ability to cope with oxidative stress under *Vibrio harveyi* and lipopolysaccharide (LPS) infection than female oysters, which provides prospects for oyster farming or oyster disease in natural seas. Gao et al. [30] investigated the effects of Streptococcus agalactiae infection on the immune and antioxidant regulatory mechanisms of golden pompano (*T. ovatus*). Results showed that *S. agalactiae* could activate TNF-α/NF-κB signaling in the liver to induce defense and immune responses. Hossen et al. [31] cloned the full length of catalase (Hdh-CAT) and found Hdh-CAT was induced by thermal stress, H_2_O_2_ induction, starvation, cadmium and immune challenges with Vibrio, lipopolysaccharides and polyinosinic–polycytidylic acid sodium salt in *P. abalone*.

We would like to acknowledge the authors that have contributed to this Special Issue “Oxidative Stress in Aquatic Organisms”. These papers offer fresh perspectives on expanding knowledge and research possibilities in the creation of antioxidant resistance in aquaculture.
